# An investigation of the effect of race-based social categorization on adults’ recognition of emotion

**DOI:** 10.1371/journal.pone.0192418

**Published:** 2018-02-23

**Authors:** B. Nicole Reyes, Shira C. Segal, Margaret C. Moulson

**Affiliations:** 1 Toronto General Hospital Research Institute, University Health Network, Toronto, Ontario, Canada; 2 Department of Psychology, Ryerson University, Toronto, Ontario, Canada; Brown University, UNITED STATES

## Abstract

Emotion recognition is important for social interaction and communication, yet previous research has identified a cross-cultural emotion recognition deficit: Recognition is less accurate for emotions expressed by individuals from a cultural group different than one’s own. The current study examined whether social categorization based on race, in the absence of cultural differences, influences emotion recognition in a diverse context. South Asian and White Canadians in the Greater Toronto Area completed an emotion recognition task that required them to identify the seven basic emotional expressions when posed by members of the same two groups, allowing us to tease apart the contributions of culture and social group membership. Contrary to our hypothesis, there was no mutual in-group advantage in emotion recognition: Participants were not more accurate at recognizing emotions posed by their respective racial in-groups. Both groups were more accurate at recognizing expressions when posed by South Asian faces, and White participants were more accurate overall compared to South Asian participants. These results suggest that in a diverse environment, categorization based on race alone does not lead to the creation of social out-groups in a way that negatively impacts emotion recognition.

## Introduction

The expression and recognition of emotions are crucial elements for social interaction. Emotion recognition has adaptive functions, with many studies reporting correlations with constructs related to adjustment, including social anxiety, academic achievement, emotional disturbance, depression, and general social competence [1–5]. Adults are generally excellent at recognizing emotions from facial expressions. However, this expertise does not apply equally to all facial expressions; there is a well-documented cross-cultural emotion recognition deficit whereby adults are less accurate at recognizing emotions expressed by individuals from different cultural backgrounds than their own (e.g., [6–12]). This has the potential to cause miscommunications, and it may be most problematic in ethnically and culturally diverse large urban centres, where individuals from different cultural backgrounds are likely to interact with each other on a daily basis.

Early studies in the field of emotion recognition emphasized the universality of emotional expression and recognition. This view posits that certain basic emotions (e.g., happiness, sadness, fear, disgust, anger, and surprise) are expressed similarly and recognized well across all cultures. Studies comparing performance on emotion judgment tasks between members of different cultures provide support for at least partial universality, in that facial expressions of basic emotions are recognized at above-chance levels in dissimilar cultural groups [13–20]. Although numerous studies have found evidence for universality of emotion recognition, these same studies have been critiqued for failing to examine cultural differences directly. More recent findings suggest cross-cultural differences in emotion expression and recognition. In a meta-analysis of 97 studies, Elfenbein et al. [21] found support for a cultural in-group advantage: Recognition performance was better when the emotions were both expressed and perceived by members of the same national, ethnic, or regional group.

The mechanisms by which this in-group advantage operates have been debated. Ekman [22] and Matsumoto [23–25] have attributed the cross-cultural emotion recognition deficit to culture-specific display rules. Display rules are the conscious use of techniques to alter the expressions of emotion according to the social norms of the culture to which one belongs (e.g., masking a negative expression to preserve social harmony; [22]). Contrary to the idea of the conscious management of emotional expressions, Elfenbein [26] posits dialect theory, which likens emotion to a universal language with different dialects in different cultures. “Dialects” in this perspective refer to subtle but consistent differences between cultures in the morphological features of particular expressions. These subtle differences in emotion *encoding* have been found to lead to decreased accuracy when recognizing emotions across cultural boundaries [9]. Emotion *decoding* is also shaped by culture. For example, Jack et al. [10] found that when viewing emotional faces, White observers look frequently to both the eyes and the mouth, whereas East Asian observers spend more time fixating the eye region. These differences in scanning behaviour led to differences in recognition accuracy: East Asian observers were more likely to confuse fearful and surprised faces, and angry and disgusted faces [10].

Critically, these mechanisms—encoding differences, decoding biases, and display rules—are thought to be the result of *culture* shaping the expression and recognition of emotion. Less attention has been paid to decoding biases that might result from social group identification, irrespective of culture. There is a wealth of literature describing in-group biases in face identity recognition, the most well studied of which is the other-race effect or own-race bias: in tasks of face perception and face memory, adults perform more poorly when recognizing or remembering faces belonging to a different race (for a review, see [27]). Similar in-group biases have been described for faces differing along dimensions of sex [28–30], age [31] and sexual orientation [32]. Two main mechanisms have been proposed to account for the in-group advantage in face recognition. The first is that the face recognition system (i.e., ‘face space’–[33]) is tuned to faces with which individuals have the most experience, leading to increased perceptual expertise with these (own-group) faces. The second is that there is increased motivation to process in-group faces, because they are perceived to be more socially relevant [34]. There is ample evidence that both mechanisms are at work in the in-group advantage in face recognition, but two lines of research give especially strong evidence for the second mechanism. The first is that an in-group advantage can be elicited through artificial group manipulations. For example, Bernstein et al. [35] randomly assigned individuals to one of two personality types (“red” or “green”), and found enhanced face recognition for supposed in-group members, and decreased recognition for supposed out-group members. The second is that faces that are racially ambiguous are recognized more accurately when they are categorized as “own-race” than when they are categorized as “other-race” [36].

While there is a large body of research demonstrating that mere social categorization can influence identity recognition, fewer studies have examined whether social categorization, in the absence of accompanying cultural biases in encoding or decoding, influence emotion recognition. This is because the majority of studies examining the cross-cultural emotion recognition deficit confound race and culture, such that participants judge emotions expressed by individuals that differ from them on both dimensions (e.g., White Americans judging East Asian faces from China). This conflation has allowed limited investigation into the contribution of social in-group/out-group processes to the cross-cultural emotion recognition deficit.

The few studies that have examined social categorization in isolation (i.e., while holding culture constant) have found mixed results. Several studies have found that when stimuli are constrained to be morphologically identical (i.e., they were coded identically using the Facial Action Coding System; [37]), such that cultural differences in the stimulus set were eliminated, the in-group advantage disappeared [9,25,38,39]. This suggests that social categorization based on race does not influence emotion recognition. However, other studies suggest an influence of social categorization on emotion recognition. For example, Young et al. [40] using the same minimal-group paradigm that they used to study identity recognition, randomly assigned individuals to one of two personality types (“red” or “green”), and found better emotion recognition for in-group faces than out-group faces, despite all faces being of the same race and cultural background as the participants. Similarly, Thibault et al. [41] found that self-reported identification with a particular group (cats in Study 1; basketball players in Study 2) led to better recognition of emotions expressed by members of that group. And a small number of studies have found that race alone does lead to an in-group advantage. For example, Kang & Lau [42] found a mutual in-group advantage when European Americans and Asian Americans rated spontaneous expressions from both groups. Tuminello et al. [43] found an in-group advantage in European American children rating posed facial expressions of European American and African American faces, but no in-group advantage for African American children rating the same faces, suggesting that the in-group advantage might also interact with a majority-group advantage.

The overall aim of the current study was to clarify the contribution of social categorization processes to the in-group advantage in emotion recognition. In particular, we were interested in whether participants would recognize facial expressions more accurately when expressed by own-race versus other-race faces, when culture was held constant. Canadians of South Asian and Western European descent completed a 7-alternative forced-choice emotion recognition task in which they identified expressions posed by unfamiliar individuals of the same two groups. As all participants in the study and all stimuli used in the study were born and raised in Canada—and thus, can be considered part of the same broader culture—this study enabled us to examine how social group membership alone, as signaled by race, affects emotion recognition. We hypothesized the following: 1) All participants would recognize happy expressions most accurately, and fearful expressions would be recognized least accurately, with the other expressions being recognized with middling accuracy. 2) A participant race by stimulus race interaction would emerge, whereby participants would recognize expressions posed by own-race individuals better than expressions posed by other-race individuals (i.e., they would demonstrate an in-group advantage). We expected that this in-group advantage might be most pronounced for negative facial expressions, because these expressions are harder to recognize overall.

## Materials and methods

### Participants

Eighty-two adults who were born and raised in Canada were recruited to participate in this study. Half of the participants were of South Asian descent (hereafter referred to as “South Asian” participants; 30 females, *M*_age_ = 19.10, *SD* = 1.50, Range: 17–23 years). Half of the participants were of Western European descent (hereafter referred to as “White” participants; 39 females, *M*_age_ = 19.37, *SD* = 2.06, Range: 18–27 years). The majority of the participants (*n* = 70) were born and raised in the Greater Toronto Area (GTA), which is highly diverse [44]. The other participants were born and raised in Ontario outside of the GTA, but all were living in the GTA at the time of testing. One South Asian participant was excluded because his data were accidentally deleted following collection. One White participant was excluded because her average emotion recognition accuracy was more than 3 standard deviations below the mean. Thus, the final sample included 40 adults in each group. A power analysis using G*Power 3 software indicated that a total sample size of 46 was required to detect a small sized effect given the statistical significance criterion of .05 [45]. Participants were recruited through a pool of undergraduate psychology students, through flyers posted on the university campus, and through online postings on free classified advertisement websites. Undergraduate psychology students received course credit and community participants received $15 for participating.

### Stimuli

To investigate our questions of interest, we created a new stimulus set. Although numerous stimulus sets have been created to investigate emotion recognition in general, and cross-cultural emotion recognition in particular, none of the existing stimulus sets contain faces of exactly the groups tested in this study (South Asian and White Canadians). Therefore, we created a new stimulus set by photographing 16 White Canadian adults (*M* = 22.31 years old, *SD* = 5.41, Range: 18–37, 10 female) and 16 South Asian Canadian adults (*M* = 22.94, *SD* = 5.04, Range: 18–37, 9 female), all of whom were born and raised in Canada.

To take the photographs, a black scarf was draped across the adult’s body so that clothing appeared uniform, and adults were asked to remove glasses and jewelry and to tie long hair back. Each adult was photographed using a Canon EOS Rebel T3i camera. Each adult posed facial expressions of anger, disgust, fear, happiness, neutral, sadness, and surprise. Studies investigating cross-cultural emotion recognition often use stimulus sets that contain faces with tightly constrained expressions of emotion. These expressions are elicited by instructing posers to move certain facial muscles in particular ways, in order to create what are considered to be highly representative versions of the underlying emotions. Several researchers (e.g., [46]) have argued that posed expressions are not representative of the emotional expressions that one encounters in everyday interactions. Additionally, studies have found that that the in-group advantage disappeared when photos of emotional expressions from different groups were constrained to have an identical appearance across cultures [9,47]. In contrast, spontaneous expressions (i.e., facial expressions elicited naturally by experiencing a particular emotion) likely capture greater cultural variations in expressive style [8,9,48]. However, it is difficult to elicit and photograph genuine emotional expressions, especially in a laboratory setting. Therefore, in the current study we sought a middle ground between tightly constrained and truly spontaneous expressions.

In order to elicit the facial expressions, the participant was asked to think of a time in their life when they felt that particular emotion and practiced expressing the emotion in a mirror in whatever way felt most natural to them. To further help the participant portray the emotion, the experimenter read scenarios intended to elicit the required emotion (see S1 Appendix for scenarios; some scenarios were created for this study and others were obtained from a previous study; [49]). No feedback was provided to the participant and the experimenter refrained from explaining how to pose emotions. Participants were given as much time as they needed until they were ready to have their picture taken.

Based on our subjective judgment, we chose the 10 best representations (5 male and 5 female) of each emotion for each of the two groups (South Asian, White). Thus, our final stimulus set consisted of 140 stimuli (2 races x 10 exemplars x 7 emotions = 140 stimuli).

### Procedure

The Research Ethics Board at Ryerson University approved this research. Informed written consent was obtained prior to testing. Because previous research has shown that making participants aware of the own-race bias can reduce or eliminate it [50], the study was initially explained as an emotion recognition study without reference to cross-cultural differences in emotion recognition.

A 7-alternative forced choice task programmed in E-Prime (Psychology Software Tools, Inc.) was used to assess recognition. Participants saw all 140 stimuli presented individually in random order on a 23” widescreen computer monitor. Participants sat approximately 55cm from the screen, such that each face subtended approximately 6.76 x 9.35 degrees of visual angle. Each face stimulus was presented in the middle of the screen against a white background with boxes containing each emotion label presented below the stimulus. Participants were instructed to choose the label that best represented the emotion expressed by each face and to respond as quickly and as accurately as possible using the mouse to click the box containing their chosen response. Each stimulus appeared on the screen until the participant made his/her response. Accuracy was recorded. After completing the task, participants were debriefed about the true purpose of the study.

## Results

### Unbiased hit rates

To assess emotion recognition accuracy, we calculated the *unbiased hit rate* (*H*_*U*_; [51]), which is the joint probability that a stimulus is correctly identified and that a response is correctly used. The unbiased hit rate corrects for response bias. If **A** is the number of times a particular type of stimulus was correctly identified, **B** is the number of times that type of stimulus was presented, and **C** is the number of times that response category was used, the unbiased hit rate is calculated as follows:

$$H_{U}=\frac{A^{2}}{\left(B\times C\right)}$$

The unbiased hit rate was calculated separately for each emotion and stimulus type (South Asian, White).

We ran a 2 (participant race: South Asian vs. White) x 2 (stimulus race: South Asian vs. White) x 7 (emotion: anger, disgust, fear, happiness, neutral, sadness, surprise) repeated-measures ANOVA on unbiased hit rates. There was the expected main effect of emotion, *F*(6, 468) = 459.73, *p* < .001, η^2^ = 0.855, with participants performing best on happy faces (H_U_ = 0.86), then neutral faces (H_U_ = 0.54), then disgusted (H_U_ = 0.48) and surprised (H_U_ = 0.43) faces, then angry (H_U_ = 0.39) and sad (H_U_ = 0.37) faces, then fearful faces (H_U_ = 0.20). There were also main effects of participant race, *F*(1, 78) = 4.90, *p* = .030, η^2^ = 0.059, and stimulus race, *F*(1, 78) = 19.12, *p* < .001, η^2^ = 0.197. White participants (H_U_ = 0.48) performed significantly better than South Asian participants (H_U_ = 0.45), and performance was significantly better for South Asian faces (H_U_ = 0.48) than White faces (H_U_ = 0.45).

**Fig 1:**
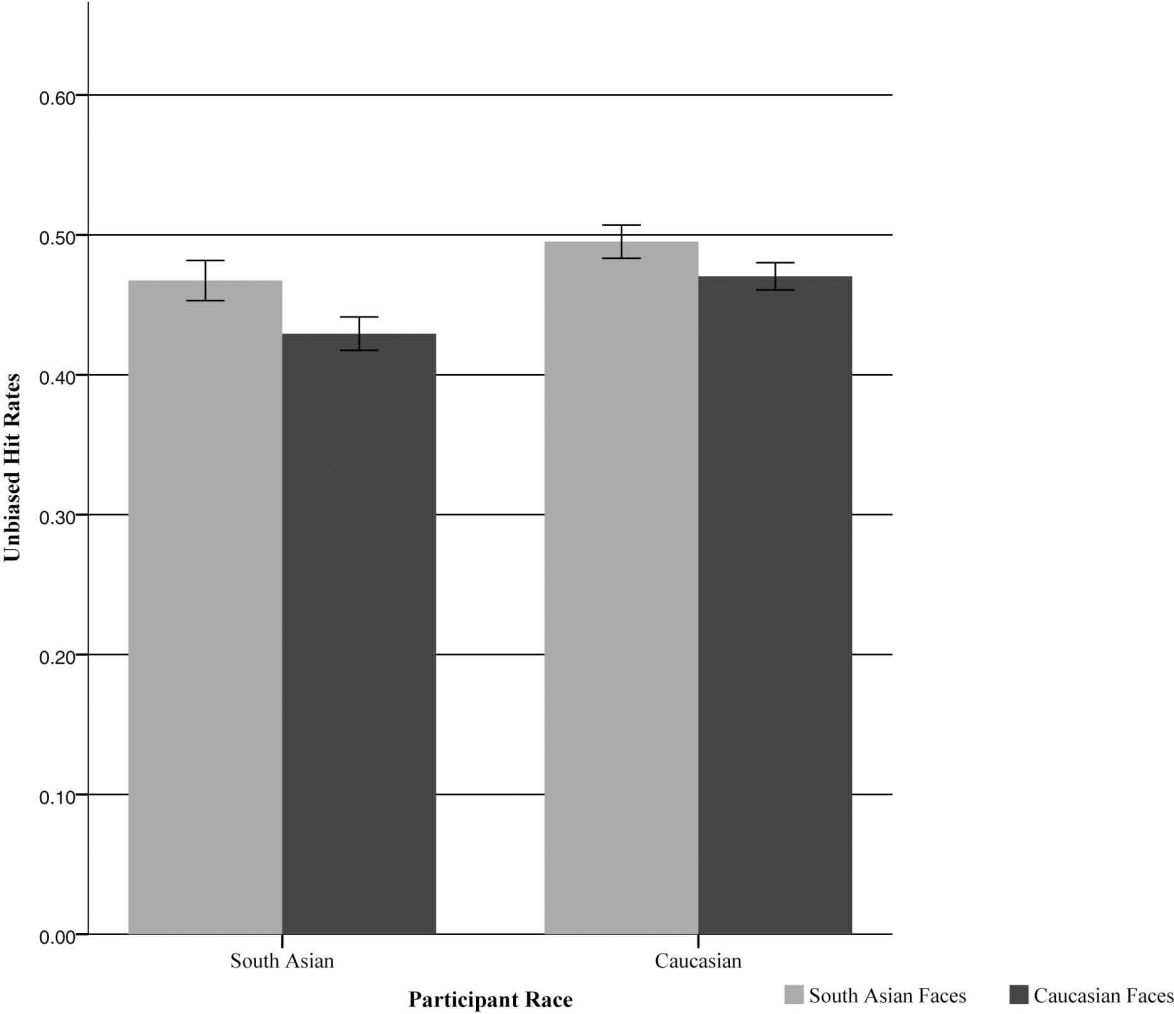
Mean accuracy for each type of face stimulus for both participant groups. There was no significant interaction between participant race and stimulus race. Error bars represent standard errors.

Contrary to our hypothesis, the critical interaction between participant race and stimulus race was not significant, *F*(1, 78) = 0.85, *p* = .359, η^2^ = 0.011 (Fig 1). However, there was a significant interaction between stimulus race and emotion, *F*(6, 468) = 13.08, *p* < .001, η^2^ = 0.144. Follow-up paired samples t-tests conducted separately for each emotion, with a Bonferroni correction for multiple comparisons, indicated that performance was better for South Asian than White angry faces (*p* < .001), fearful faces (*p* < .001), happy faces (*p* < .001), and surprised faces (*p* = .004); performance was equivalent for South Asian and White neutral faces (*p* = .894) and sad faces (*p* = .992); and performance was better for White than South Asian disgusted faces (*p* = .001).

### Misidentification errors

To analyze misidentification errors we first examined confusion matrices for South Asian (Table 1) and White (Table 2) stimuli. Since we were interested in whether the different participant groups differed in their likelihood of confusing particular emotions (e.g., mistakenly labeling anger as disgust), we calculated odds ratios for each of the incorrect responses. There were no significant differences between the participant groups for any of the errors made for either White or South Asian faces. Thus, White and South Asian participants did not differ in the particular patterns of confusion errors that they made.

**Table 1:** Confusion matrix for South Asian faces.

Stimulus	Judgment													
	Anger		Disgust		Fear		Happiness		Neutral		Sadness		Surprise	
	White	South Asian	White	South Asian	White	South Asian	White	South Asian	White	South Asian	White	South Asian	White	South Asian
Anger	**58.00**	**55.50**	12.50	12.50	4.75	7.25	0	0	13.50	12.25	10.25	8.25	1.00	4.25
Disgust	10.50	11.50	**60.75**	**59.25**	4.00	4.75	7.50	8.00	4.00	3.50	12.25	10.00	1.00	3.00
Fear	3.25	2.00	8.50	9.50	**36.75**	**33.00**	0.25	0	3.75	3.00	1.00	1.00	46.50	51.50
Happiness	0	0	0	0	0	0	**100**	**99.00**	0	0.75	0	0	0	0.25
Neutral	3.25	2.00	0.25	0.50	0.75	1.75	0	1.25	**91.50**	**89.50**	4.25	4.25	0	0.75
Sadness	3.00	3.75	1.50	1.75	1.00	3.75	0.25	0.50	38.00	39.25	**55.00**	**49.50**	1.25	1.50
Surprise	0.25	0.50	2.00	0.75	9.75	9.75	1.75	3.25	8.75	8.50	1.25	0.75	**76.25**	**76.50**

*Note*. Expected response is bolded.

**Table 2:** Confusion matrix for White faces.

Stimulus	Judgment													
	Anger		Disgust		Fear		Happiness		Neutral		Sadness		Surprise	
	White	South Asian	White	South Asian	White	South Asian	White	South Asian	White	South Asian	White	South Asian	White	South Asian
Anger	**57.25**	**48.25**	10.00	12.75	0.50	3.00	0.50	1.00	17.00	19.00	14.00	12.50	0.75	3.50
Disgust	19.25	15.25	**62.00**	**65.25**	3.00	2.25	0	0.75	1.25	1.50	8.50	7.75	6.00	7.25
Fear	1.75	5.00	4.50	4.50	**30.00**	**20.00**	0.50	1.75	18.75	16.75	3.25	2.00	41.25	50.00
Happiness	0	0	0	0	0	0	**98.50**	**97.00**	1.50	2.50	0	0	0	0.50
Neutral	3.25	1.25	0.25	0	0.50	2.00	1.25	1.25	**92.00**	**92.00**	2.75	3.25	0	0.25
Sadness	10.75	8.50	3.75	1.00	1.75	4.75	0	0	27.25	34.75	**56.00**	**50.50**	0.50	0.50
Surprise	0	0	0.25	0	11.00	10.25	15.50	18.50	0.50	1.25	0	0	**72.75**	**70.00**

*Note*. Expected response is bolded.

**Fig 2:**
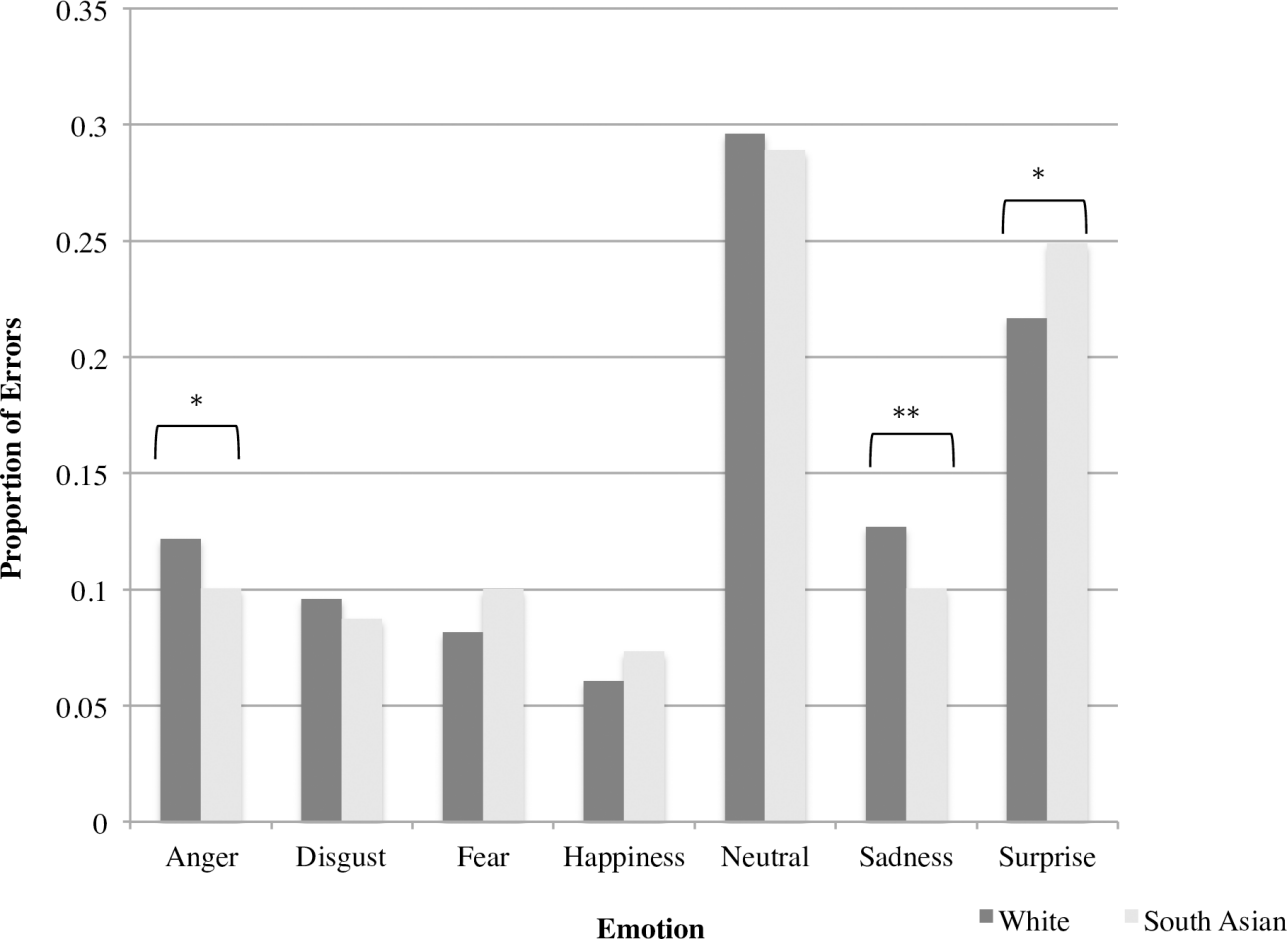
Proportion of errors for White and South Asian Canadians across all stimuli. *p < .05; **p < .02.

**Fig 3:**
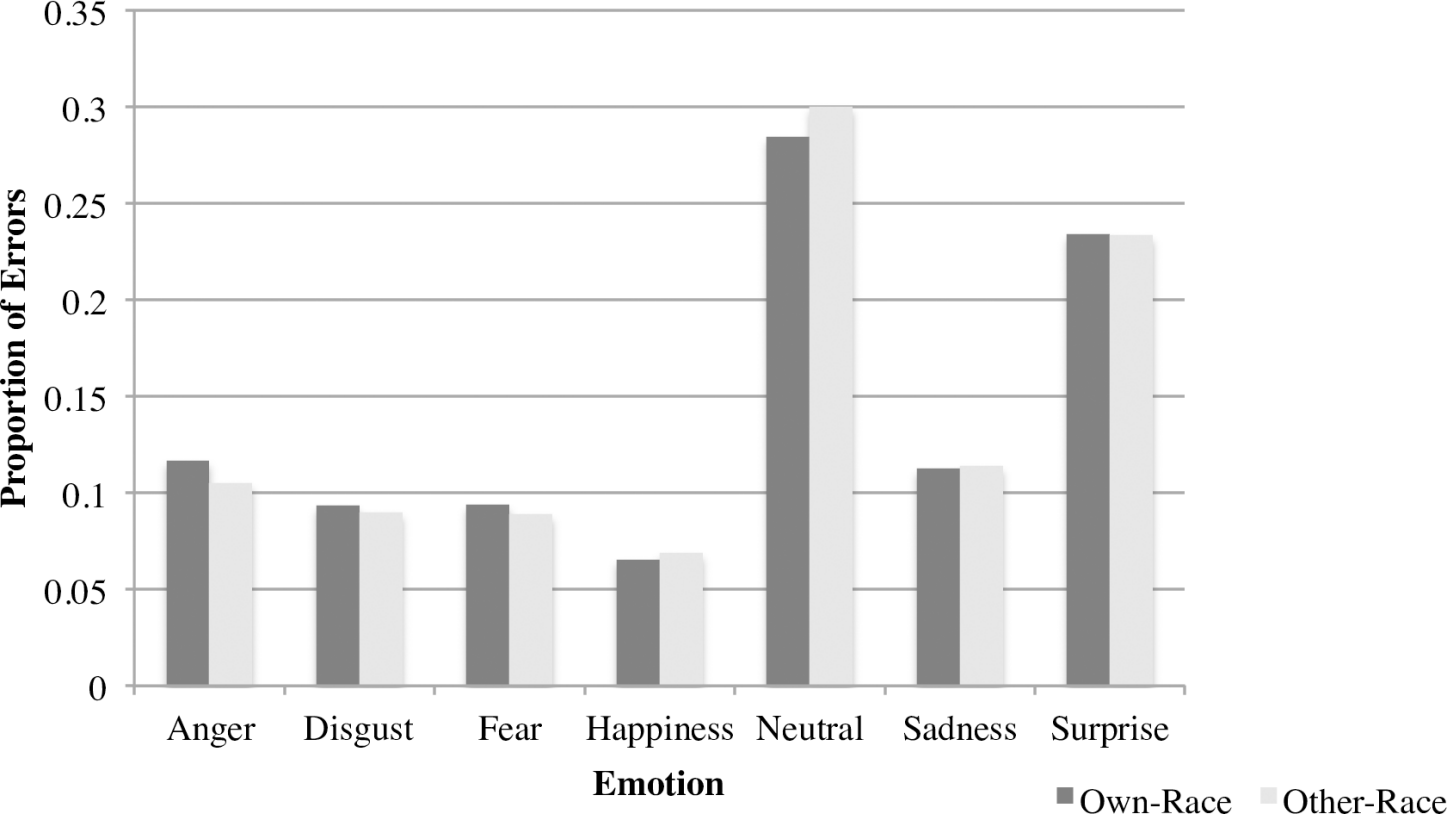
Proportion of errors for own-race faces (White group judging White faces and South Asian group judging South Asian faces) and other-race faces (White group judging South Asian faces and South Asian faces judging White faces). There were no significant differences between groups on any emotions.

We then analyzed overall error patterns using chi-square tests to determine if participant groups differed in their likelihood of attributing particular emotions, regardless of the emotion portrayed by the stimulus (e.g., an increased likelihood of attributing anger across all stimulus faces). Collapsing across White and South Asian stimuli, White participants over-attributed anger (χ^2^ = 3.89, *p* < .05) and sadness (χ^2^ = 5.79, *p* < .02), and under-attributed surprise (χ^2^ = 4.24, *p* < .05) compared to South Asian participants (Fig 2). Thus, there were some differences between the groups in their likelihood of attributing some emotions. To determine whether attributions differed for own-race compared to other-race faces, we used chi-square tests to compare the error responses made for own-race compared to other-race faces, collapsed across participant groups. There were no significant differences in the attributions made for own-race compared to other-race faces (Fig 3). Thus, the differences between White and South Asian participants in their attribution of anger, sadness, and surprise did not differ depending on whether faces were of their own or another race.

## Discussion

The goal of this study was to investigate whether social categorization based on race influenced emotion recognition when culture was held constant. South Asian and White Canadians completed an emotion recognition task with stimuli from the same two groups. The major finding that emerged from this study was that, contrary to our hypothesis, there was no evidence of an in-group advantage in emotion recognition accuracy; participants did not show better recognition accuracy for own-race than other-race faces. We did find main effects of encoder group and decoder group: Overall, performance was better on South Asian faces than on White faces, and White participants were more accurate than South Asian participants. The pattern of errors revealed an effect of decoder group, as well: White participants were more likely to attribute anger and sadness to emotional faces, whereas South Asian participants were more likely to attribute surprise. As predicted, participants were most accurate at recognizing happy faces and least accurate at recognizing fearful faces.

That there was no in-group advantage in emotion recognition accuracy was unexpected. Previous studies have found that social categorization based on race [42,43] and other natural groups (e.g., identification with basketball players; [41]) influences emotion recognition. And perhaps the strongest evidence that social categorization influences emotion recognition is that assigning people to artificial groups leads to better emotion recognition for faces that supposedly belong to the same group vs. faces that supposedly belong to a different group [40]. There is also abundant evidence that social categorization, particularly based on race, influences identity recognition.

However, our results are in line with other studies showing no in-group advantage based on race when cultural differences between emotional expressions of different ethnic groups are erased by using standardized stimuli. For example, Elfenbein et al. [9] found that Québecois and Gabonese participants showed an in-group advantage when judging spontaneous facial expressions, but *not* when judging stimuli from the same ethnic groups that had been standardized to erase cultural differences. In the current study, we did not intentionally erase cultural differences in the stimuli. Rather, we anticipated that there would be few, if any, systematic cultural differences in the expression or decoding of the emotions in this study because all of the expressers and perceivers in this study were from the same broader cultural background (Canadian). Using a similar approach, Prado et al. [52] found no differences in performance between people of Chinese heritage living in Australia and White Australians on an emotion recognition task of Chinese and White stimuli. As in our study, when culture was held constant race alone did not appear to lead to an in-group advantage in emotion recognition.

Another possible explanation for the lack of an in-group advantage in the current study is the specific population that was tested. All of our participants were living in the Greater Toronto Area at the time of testing and the majority of participants were born and raised in the GTA. The GTA is highly diverse: Visible minorities make up almost half of the population (47.1%; [44]) and South Asians are the largest minority group (15.1%; [44]). It is possible that growing up in the GTA might buffer against an in-group advantage in emotion recognition because race may not actually signal in-group or out-group membership to our participants. Or perhaps race is a signal of social group membership, but one that does not lead to the decreased motivation to process out-group faces that is traditionally observed. This possible explanation could be further explored in studies that include a measure of inter-group contact (as in [53]) and/or a measure of racial bias, such as the Implicit Association Task [54]. The current study did not include either measure, and thus we can only speculate about the effect that living in a diverse city may have on the exposure to own- versus other-race individuals, the saliency of in-group/out-group categorization based on race, and the motivation to decode faces of out-group members. Individuation training of other-race faces has previously been found to reduce implicit racial bias in adults [55], suggesting a relationship between the social and perceptual processing of other-race faces. Future studies should include measures of inter-group contact and racial bias to explore this relationship with respect to emotion recognition.

Consistent with this possibility, there is evidence that the other-race effect in identity recognition becomes smaller as individuals become more familiar with other-race faces, either through natural experience (e.g., Chinese individuals living in Australia showed an other-race effect for White faces that decreased in size in proportion to the length of time living in Australia; [53] or experimental manipulation (e.g., infants given experience individuating other-race faces show a maintained ability to discriminate other-race faces; [56]. There is also evidence that the cross-cultural recognition deficit is smaller as groups get closer geographically [21], although this is usually interpreted as indicating that cultural variations in expressive style are more similar for geographically closer populations.

To determine whether these findings are specific to participants living in highly diverse cities like the GTA, it would be interesting to test a group of White participants from a more homogenous town in Ontario with the same stimulus set. Again, cultural differences in encoding and decoding would be controlled (since all stimulus faces and participants would be from the same cultural background–Canadian), but the decoding population in this case might be more likely to see the South Asian faces as representing an out-group, and thus might be more likely to show decreased accuracy on South Asian compared to White faces.

We did find main effects of encoding group and decoding group. Participants were more accurate overall when rating South Asian than White faces (an encoder effect). It has been suggested that physiognomy may play an important role in emotion recognition, such that certain expressions may be more or less difficult to decode depending on actual facial morphology [38]. For example, the furrowed brow that is characteristic of angry expressions might be easier to detect in individuals with prominent eyebrows. In the current study, perhaps South Asian expressions were easier to detect due to differences in facial, rather than expressive, morphology.

In terms of decoding, the White participants showed better performance overall than the South Asian participants, although it should be noted that this was a small effect. There were also somewhat different patterns of errors between the two groups, with White participants being more likely to attribute anger and sad, and South Asian participants being more likely to attribute surprise to the emotional faces. These decoding biases are potentially consistent with previous research showing that individuals from individualistic cultures are more likely to report seeing negative emotion than individuals in collectivistic cultures (e.g., [25]). Although all of our participants were born and raised in Canada, it is possible that there were still differences in their immediate environments that manifested in decoding biases. Specifically, our South Asian participants were almost all second-generation Canadians (i.e., one or both parents were born outside Canada). Thus, their family environments might reflect more collectivist values than the family environments of our White participants. These differences in micro-culture, even against a backdrop of the same wider culture, might have led to the decreased likelihood of our South Asian participants to report negative emotion. Importantly, these decoding biases did not differ for in-group versus out-group faces–i.e., participants did not show different decoding biases for own-race vs. other-race faces–which lends further support to our conclusion that that there was no in-group advantage in emotion recognition for own-race faces in the current study.

In summary, the current study found that social categorization based on race, in the absence of accompanying cultural differences, did not lead to decreased emotion recognition accuracy. Future studies should examine this phenomenon in more homogenous populations, where race might be a stronger signal for in-group vs. out-group membership. Additionally, few studies have examined whether other naturally-occurring signals of group membership (e.g., age) lead to differences in emotion recognition accuracy. This study adds to the literature on the factors that influence emotion recognition across ethnic and cultural boundaries.

## Supporting information

S1 Appendix Emotion scenarios. (DOCX)

## References

[1] Eisenberg N, Fabes RA, Guthrie IK, Murphy BC, Maszk P, Holmgren R, et al The relations of regulation and emotionality to problem behavior in elementary school children. Dev Psychopathol. 1996;8: 141–162. doi: 10.1017/S095457940000701X

[2] Izard CE. The face of emotion Century psychology series. New York: Appleton-Century-Crofts; 1971.

[3] McClure EB, Nowicki S. Associations between social anxiety and nonverbal processing skill in preadolescent boys and girls. J Nonverbal Behav. 2001;25: 3–19. doi: 10.1023/A:1006753006870

[4] Nowicki S, Carton E. The relation of nonverbal processing ability of faces and voices and children’s feelings of depression and competence. J Genet Psychol. 1997;158: 357–363. doi: 10.1080/00221329709596674 9255962 10.1080/00221329709596674

[5] Yoo SH, Matsumoto D, LeRoux JA. The influence of emotion recognition and emotion regulation on intercultural adjustment. Int J Intercult Relations. 2006;30: 345–363. doi: 10.1016/j.ijintrel.2005.08.006

[6] Dailey MN, Joyce C, Lyons MJ, Kamachi M, Ishi H, Gyoba J, et al Evidence and a computational explanation of cultural differences in facial expression recognition. Emotion. 2010;10: 874–893. doi: 10.1037/a0020019 21171759 10.1037/a0020019 PMC7360061

[7] Elfenbein HA, Ambady N. When familiarity breeds accuracy: Cultural exposure and facial emotion recognition. J Pers Soc Psychol. 2003;85: 276–290. doi: 10.1037/0022-3514.85.2.276 12916570 10.1037/0022-3514.85.2.276

[8] Elfenbein HA, Ambady N. Universals and cultural differences in recognizing emotions. Curr Dir Psychol Sci. 2003;12: 159–164. doi: 10.1111/1467-8721.01252

[9] Elfenbein HA, Beaupre M, Levesque M, Hess U. Toward a dialect theory: Cultural differences in the expression and recognition of posed facial expressions. Emotion. 2007;7: 131–146. doi: 10.1037/1528-3542.7.1.131 17352569 10.1037/1528-3542.7.1.131

[10] Jack RE, Blais C, Scheepers C, Schyns PG, Caldara R. Cultural confusions show that facial expressions are not universal. Curr Biol. 2009;19: 1543–1548. doi: 10.1016/j.cub.2009.07.051 19682907 10.1016/j.cub.2009.07.051

[11] Yan X, Andrews TJ, Jenkins R, Young AW. Cross-cultural differences and similarities underlying other-race effects for facial identity and expression. Q J Exp Psychol. 2016;69: 1247–1254. doi: 10.1080/17470218.2016.1146312 26878095 10.1080/17470218.2016.1146312

[12] Yan X, Andrews TJ, Young AW. Cultural similarities and differences in perceiving and recognizing facial expressions of basic emotions. J Exp Psychol Hum Percept Perform. 2016;42: 423–440. doi: 10.1037/xhp0000114 26480247 10.1037/xhp0000114

[13] Boucher JD, Carlson GE. Recognition of facial expression in three cultures. J Cross Cult Psychol. 1980;11: 263–280. doi: 10.1177/0022022180113003

[14] Ekman P. Strong evidence for universals in facial expressions: A reply to Russell’s mistaken critique. Psychol Bull. American Psychological Association; 1994;115: 268–287. doi: 10.1037/0033-2909.115.2.268 8165272 10.1037/0033-2909.115.2.268

[15] Ekman P, Friesen W V., O’Sullivan M, Chan A, Diacoyanni-Tarlatzis I, Heider K, et al Universals and cultural differences in the judgements of facial expressions of emotion. J Pers Soc Psychol. 1987;53: 712–717. 3681648 10.1037//0022-3514.53.4.712

[16] Ekman P, Sorenson ER, Friesen W V. Pan-cultural elements in facial displays of emotion. Science 1969;164: 86–88. doi: 10.1126/science.164.3875.86 5773719 10.1126/science.164.3875.86

[17] Ekman P, Friesen W V. Constants across cultures in the face and emotion. J Pers Soc Psychol. 1971;17: 124–129. doi: 10.1037/h0030377 5542557 10.1037/h0030377

[18] Jeon B, Landgrebe DA. Absolute classification with unsupervised clustering. International Geoscience and Remote Sensing Symposium (IGARSS). 1992 1609–1611. doi: 10.1109/IGARSS.1992.578647

[19] Matsumoto D, Olide A, Schug J, Willingham B, Callan M. Cross-cultural judgments of spontaneous facial expressions of emotion. J Nonverbal Behav. 2009;33: 213–238. doi: 10.1007/s10919-009-0071-4

[20] McAndrew FT. A Cross-cultural study of recognition thresholds for facial expressions of emotion. J Cross Cult Psychol. 1986;17: 211–224. doi: 10.1177/0022002186017002005

[21] Elfenbein HA, Ambady N. On the universality and cultural specificity of emotion recognition: A meta-analysis. Psychol Bull. 2002;128: 203–235. doi: 10.1037/0033-2909.128.2.203 11931516 10.1037/0033-2909.128.2.203

[22] Ekman P. Universals and cultural differences in facial expressions of emotion. Nebraska Symposium on Motivation. 1972 207–283. doi: 10.1037/0022-3514.53.4.712

[23] Matsumoto D. Cultural Influences on the perception of emotion. J Cross Cult Psychol. 1989;20: 92–105. doi: 10.1177/0022022189201006

[24] Matsumoto D. Cultural similarities and differences in display rules. Motiv Emot. 1990;14: 195–214. doi: 10.1007/BF00995569

[25] Matsumoto D. American-Japanese cultural differences in the recognition of universal facial expressions. J Cross Cult Psychol. 1992;23: 72–84. doi: 10.1177/0022022192231005

[26] Elfenbein HA. Nonverbal dialects and accents in facial expressions of emotion. Emot Rev. 2013;5: 90–96. doi: 10.1177/1754073912451332

[27] Meissner CA, Brigham JC. Thirty years of investigating the own-race bias in memory for faces: A meta-analytic review. Psychol Public Policy, Law. American Psychological Association; 2001;7: 3–35. doi: 10.1037//1076-8971.7.1.3

[28] Lewin C, Herlitz A. Sex differences in face recognition—women’s faces make the difference. Brain Cogn. 2002;50: 121–128. doi: 10.1016/S0278-2626(02)00016-7 12372357 10.1016/s0278-2626(02)00016-7

[29] Lovén J, Herlitz A, Rehnman J. Women’s own-gender bias in face recognition memory: The role of attention at encoding. Exp Psychol. 2011;58: 333–340. doi: 10.1027/1618-3169/a000100 21310695 10.1027/1618-3169/a000100

[30] Wolff N, Kemter K, Schweinberger SR, Wiese H. What drives social in-group biases in face recognition memory? ERP evidence from the own-gender bias. Soc Cogn Affect Neurosci. 2014;9: 580–590. doi: 10.1093/scan/nst024 23474824 10.1093/scan/nst024 PMC4014097

[31] Rhodes MG, Anastasi JS. The own-age bias in face recognition: A meta-analytic and theoretical review. Psychol Bull. 2012;138: 146–174. doi: 10.1037/a0025750 22061689 10.1037/a0025750

[32] Rule NO, Ambady N, Adams RB, Macrae CN. Us and them: Memory advantages in perceptually ambiguous groups. Psychon Bull Rev. 2007;14: 687–692. doi: 10.3758/BF03196822 17972734 10.3758/bf03196822

[33] Valentine T. A unified account of the effects of distinctiveness, inversion, and race in face recognition. Q J Exp Psychol Sect A Hum Exp Psychol. 1991;43: 161–204. doi: 10.1080/14640749108400966 10.1080/14640749108400966 1866456

[34] Sporer SL. Recognizing faces of other ethnic groups An integration of theories. Psychol Public Policy, Law. 2001;7: 36–97. doi: 10.1037/1076-8971.7.1.36

[35] Bernstein MJ, Young SG, Hugenberg K. The cross-category effect. Psychol Sci. 2007;18: 706–712. doi: 10.1111/j.1467-9280.2007.01964.x 17680942 10.1111/j.1467-9280.2007.01964.x

[36] MacLin OH, Malpass RS. Racial categorization of faces: The ambiguous race face effect. Psychol Public Policy, Law. 2001;7: 98–118. doi: 10.1037/1076-8971.7.1.98

[37] Ekman P, Friesen W V. The facial action coding system. Consulting. 1978;

[38] Beaupre MG, Hess U. Cross-cultural emotion recognition among Canadian ethnic groups. J Cross Cult Psychol. 2005;36: 355–370. doi: 10.1177/0022022104273656

[39] Biehl M, Matsumoto D, Ekman P, Hearn V, Heider K, Kudoh T, et al Matsumoto and Ekman’s Japanese and Caucasian facial expressions of emotion (JACFEE): Reliability data and cross-national differences. J Nonverbal Behav. 1997;21: 3–21. doi: 10.1023/a:1024902500935

[40] Young SG, Hugenberg K. Mere social categorization modulates identification of facial expressions of emotion. J Pers Soc Psychol. 2010;99: 964–977. doi: 10.1037/a0020400 20919774 10.1037/a0020400

[41] Thibault P, Bourgeois P, Hess U. The effect of group-identifcation on emotion recognition: The case of cats and basketball players. J Exp Soc Psychol. 2006;42: 676–683. doi: 10.1016/j.jesp.2005.10.006

[42] Kang S-M, Lau AS. Revisiting the out-group advantage in emotion recognition in a multicultural society: Further evidence for the in-group advantage. Emotion. 2013;13: 203–215. doi: 10.1037/a0030013 23163711 10.1037/a0030013 PMC7055482

[43] Tuminello ER, Davidson D. What the face and body reveal: In-group emotion effects and stereotyping of emotion in African American and European American children. J Exp Child Psychol. 2011;110: 258–274. doi: 10.1016/j.jecp.2011.02.016 21444092 10.1016/j.jecp.2011.02.016

[44] Statistics Canada. National Household Survey (NHS) Profile, 2011. In: Toronto, CMA, Ontario (Code 535). 2013.

[45] Faul F, ErdFelder E, Lang A-G, Buchner A. G*Power 3.1 manual. Behav Res Methods. 2007;39: 175–191. doi: 10.3758/BF03193146 17695343 10.3758/bf03193146

[46] Naab PJ, Russell JA. Judgments of emotion from spontaneous facial expressions of New Guineans. Emotion. 2007;7: 736–744. doi: 10.1037/1528-3542.7.4.736 18039042 10.1037/1528-3542.7.4.736

[47] Beaupré MG, Hess U. Cross-cultural emotion recognition among Canadian ethnic groups. J Cross Cult Psychol. 2005;36: 355–370. doi: 10.1177/0022022104273656

[48] Jack RE, Caldara R, Schyns PG. Internal representations reveal cultural diversity in expectations of facial expressions of emotion. J Exp Psychol Gen. 2012;141: 19–25. doi: 10.1037/a0023463 21517206 10.1037/a0023463

[49] Jones AP, Happé FGE, Gilbert F, Burnett S, Viding E. Feeling, caring, knowing: Different types of empathy deficit in boys with psychopathic tendencies and autism spectrum disorder. J Child Psychol Psychiatry. 2010;51: 1188–1197. doi: 10.1111/j.1469-7610.2010.02280.x 20633070 10.1111/j.1469-7610.2010.02280.x PMC3494975

[50] Hugenberg K, Miller J, Claypool HM. Categorization and individuation in the cross-race recognition deficit: Toward a solution to an insidious problem. J Exp Soc Psychol. 2007;43: 334–340. doi: 10.1016/j.jesp.2006.02.010

[51] Wagner HL. On measuring performance in category judgment studies of nonverbal behavior. J Nonverbal Behav. 1993;17: 3–28. doi: 10.1007/BF00987006

[52] Prado C, Mellor D, Byrne LK, Wilson C, Xu X, Liu H. Facial emotion recognition: A cross-cultural comparison of Chinese, Chinese living in Australia, and Anglo-Australians. Motiv Emot. 2014;38: 420–428. doi: 10.1007/s11031-013-9383-0

[53] Hancock KJ, Rhodes G. Contact, configural coding and the other-race effect in face recognition. Br J Psychol. 2008;99: 45–56. doi: 10.1348/000712607X199981 17535471 10.1348/000712607X199981

[54] Greenwald AG, Greenwald AG, Mcghee DE, Mcghee DE, Schwartz JLK, Schwartz JLK. Measuring individual differences in implicit cognition: The implicit association test. J Personal Soclal Psychol. 1998;74: 1464–1480. doi: 10.1037/0022-3514.74.6.1464 10.1037//0022-3514.74.6.1464 9654756

[55] Lebrecht S, Pierce LJ, Tarr MJ, Tanaka JW. Perceptual other-race training reduces implicit racial bias. PLoS One. 2009;4 doi: 10.1371/journal.pone.0004215 19156226 10.1371/journal.pone.0004215 PMC2627769

[56] Heron-Delaney M, Anzures G, Herbert JS, Quinn PC, Slater AM, Tanaka JW, et al Perceptual training prevents the emergence of the other race effect during infancy. PLoS One. 2011;6 doi: 10.1371/journal.pone.0019858 21625638 10.1371/journal.pone.0019858 PMC3097220

