# Supplementary material for: An investigation of the effect of race-based social categorization on adults’ recognition of emotion
Source: PLoS One. 2018 Feb 23;13(2):e0192418. doi: 10.1371/journal.pone.0192418 (PMC5825022; doi:10.1371/journal.pone.0192418)
Supplement: S1 Appendix — (DOCX) [file pone.0192418.s001.docx]

**S1 Appendix**

**S1 Appendix. Emotion Scenarios.**

| Emotion | Scenarios |
| --- | --- |
| Happiness | You just received a promotion at work! You are going on your dream vacation! You have won a contest to win an iPod! |
| Angry | While doing a group project, someone took credit for your great work. Someone bumped into your car in the parking lot and did not leave a note with their information.  Your best friend reveals your secrets to others. |
| Sadness | You’ve applied for a job that you really wanted and are qualified for and have just discovered that you have not been selected for the job.  Your favourite grade school teacher passed away.  Your plane got delayed and you’re going to miss your best friend's wedding. |
| Fear | [If participant is a woman] A strange man’s face appeared at your window in the night.  [If participant is male] A strange face appeared at your window in the night.  You were riding your bike down a hill, when suddenly your brakes stopped working. |
| Surprise | A man on a motorbike suddenly swerved and almost hit you.   You open the door and someone is unexpectedly on the other side.  You come home and find that your roommate has repainted the rooms.  You walk inside your home and find that your friends threw you a party and yell ‘HAPPY BIRTHDAY!!’ |
| Disgust | You are eating lunch and you find a cockroach in your sandwich.  You go to wash the dishes and find maggots in the sink.  Someone had left the toilet seat dirty and you sat on it by mistake. |
